# Comprehensive studies on the properties of apple juice treated by non-thermal atmospheric plasma in a flow-through system

**DOI:** 10.1038/s41598-020-78131-6

**Published:** 2020-12-03

**Authors:** Anna Dzimitrowicz, Aleksandra Bielawska-Pohl, Pawel Pohl, Piotr Cyganowski, Agata Motyka-Pomagruk, Tymoteusz Klis, Malgorzata Policht, Aleksandra Klimczak, Piotr Jamroz

**Affiliations:** 1 Department of Analytical Chemistry and Chemical Metallurgy, Wroclaw University of Science and Technology, Wybrzeze St. Wyspianskiego 27, 50-370 Wroclaw, Poland; 2 Laboratory of Biology of Stem and Neoplastic Cells, Hirszfeld Institute of Immunology and Experimental Therapy Polish Academy of Science, R. Weigla 12, 53-114 Wroclaw, Poland; 3 Department of Polymer and Carbonaceous Materials, Wroclaw University of Science and Technology, Wybrzeze St. Wyspianskiego 27, 50-370 Wroclaw, Poland; 4 Intercollegiate Faculty of Biotechnology, and Medical University of Gdansk, Laboratory of Plant Protection and Biotechnology, University of Gdansk, Abrahama 58, 80-307 Gdansk, Poland

**Keywords:** Electrical and electronic engineering, Nutrition, Applied microbiology

## Abstract

We present an optimized non-thermal atmospheric plasma (NTAP)-based reaction-discharge system that was applied for a continuous-flow treatment of apple juice (AJ). To optimize this system for a high-throughput production of AJ with ameliorated properties, the effect of several parameters was studied using design of experiments approach followed by the response surface methodology. Additionally, nutritional, physicochemical, microbiological and cytotoxic properties of resulting AJ were assessed. It was established that NTAP treatment of AJ led to rise in concentration of Ca, Fe, K, Mg, Na and Sr by 8–10% as well as Al, B, Ba, Cu, Mn and Zn by 11–15%. Additionally, the increased total phenolic content by ~ 11% in addition to the prolonged by up to 12 days shelf life of the product were observed. Moreover, it was found that the NTAP-treatment of AJ did not change the structure of organic compounds present in AJ, in addition to its °Brix value, color and ferric ion reducing antioxidant power. Furthermore, AJ subjected to NTAP did not show any cytotoxic activity towards non-malignant human intestinal epithelial cells but exhibited induced cell cytotoxicity in human colorectal adenocarcinoma cells. Our study provided arguments for future introduction of these types of preparations to the global market.

## Introduction

Apple, being a national Polish bijou, is a frequently grown and consumed fruit in the whole Europe and beyond. Only in 2013, Poland has exported 1,230 thousand tons of apples abroad, which made it a trade leader of this fruit^1^. Popularity of apples is associated with their excellent taste as well as high nutritional properties^1^. By now, several scientific groups focused on amelioration of beneficial properties of apples with the use of non-thermal atmospheric plasmas (NTAPs). In this case, NTAPs were applied to treat apple juices (AJs)^2–8^ or the fresh fruit mass^9,10^. AJ is willingly consumed and this is associated with its advantageous effects on health, mostly due to the presence of organic compounds (such as e.g., vitamins^2^ and polyphenols^3^) as well as major and minor elements (such as e.g., Ca, Fe, K, Mg and Na^11^).

Because NTAPs are rich sources of reactive oxygen species (ROS) and reactive nitrogen species (RNS)^12^, NTAP-treated products of apple-origin exhibit different properties in contrast to untreated ones^2–10^. Among physicochemical properties of NTAP-treated AJs, color^2–4,7^, the total polyphenol content^2–4^, non-enzymatic browning^3^, titratable acidity^4,7^, the ^o^Brix value^4^, cloud stability^3^, polyphenol oxidase activity^3^, the cell membrane potential^5^, the total soluble solid content^7^, and pH^2–4,7^ were previously examined. In terms of microbiological properties of NTAP-treated AJs, application of NTAPs led to inactivation of microorganisms belonging to several diverse species, *e.g.*, *Escherichia coli*^2,4,9^, *Citrobacter freundii*^5^, *Zygosaccharomyces rouxii*^6–8^, *Salmonella typhimurium*^9^ and *Salmonella choleraesuis*^9^, which resulted in prolongation of the shelf life of such AJs and ensured their higher food safety and quality. Regarding cytotoxic activity of such preparations, only NTAP-treated fresh apples^10^ were suggested so far not to pose any danger for human health.

It is also worth noticing that all NTAP-based reaction-discharge systems, previously used to treat AJs^2–8^, worked in a stationary mode, which limited the volume of the processed liquid. Unfortunately, in such non-flow-through NTAP-based stationary reaction-discharge systems, there could appear some problems associated with uncontrolled plasma-liquid interactions. Besides, a narrow range of NTAP types and reaction-discharge systems was used so far to expose AJs to effects of plasma processes and reactions, i.e., devices based on an atmospheric pressure plasma jet^2^, spark discharge^3^, glow discharge^3^, dielectric barrier discharge^4,6–8^ or a commercially available kINPen09 instrument^5^. One should also have in mind that only the one-factor-at-a-time (OFAT) approach was applied to examine the impact of the NTAP treatment on AJs in terms of their resultant properties^2–7^, therefore not effectively determining the influence of two or more parameters and their interactions on final features of these AJs.

To cope with these difficulties, a new high-throughput NTAP-based reaction-discharge system, intended for a continuous-flow treatment of fruit and vegetables juices^13^, was developed in our research group. Here, to have control on the course of interactions of NTAP with studied AJ and its most desirable properties, including, color, taste and aroma, a multiparameter optimization of selected operating parameters of the proposed flow-through NTAP system by the design of experiments (DOE) approach was carried out and followed by the response surface methodology (RSM)^14^. To the best of our knowledge, there is no work, in which such approach has been undertaken. Furthermore, there are no studies, in which the concentration of elements in NTAP-treated AJ was determined while its changes were discussed. We hypothesize that the treatment of AJ in the NTAP flow-through reaction-discharge system, controlled by the DOE approach, will improve the nutritional value, shelf life and quality of this product. For that reason, the main objective of this work was to optimize the above-mentioned high-throughput NTAP-based continuous-flow reaction-discharge system intended for the controlled treatment of AJ. The effect of selected parameters such as the discharge current, the flow rate of AJ, and the distance between the electrodes on the absorbance (A) of the most prominent absorption band in the UV/Vis spectrum of AJ and its temperature (T) were simultaneously studied. Both responses were fitted with appropriate response surface regression models, which enabled to select such settings of studied parameters that provided the unchanged A value of the absorption band identified in the spectrum of plasma-treated AJ and its higher T (but not higher than 50 °C). These criteria for both responses were selected to avoid degrading the chemical composition of plasma-treated AJ, while prolonging its shelf life and biological safety. Afterwards, NTAP-treated AJ, obtained by operating the flow-through FLC-dc-APGD reaction-discharge system under optimal conditions, was subjected to a series of analyses associated with evaluation of its nutritional, physicochemical as well microbiological properties. In addition, cytotoxicity of resulting plasma-treated AJ towards the normal human intestinal epithelial cell line (FHs 74 Int) and human colorectal adenocarcinoma cells (Caco-2) was assessed.

## Methods

### Reagents and solutions

Commercially available AJ was used in all experiments. Before the NTAP treatment, AJ was filtered through a qualitative hard filter paper (150 mm; Chemland, Poland). To perform the Folic-Ciocalteu (F–C) test for phenolic species, the Folin & Ciocalteu’s phenol reagent, anhydrous sodium carbonate and gallic acid monohydrate were obtained from Sigma-Aldrich (Germany). To carry out the ferric ion reducing antioxidant power (FRAP) assay, 2,4,6-tis(2-pirydyl)-S-traizine (TPTZ), ferric sulphate (FeSO_4_), ferric chloride hexahydrate (FeCl_3_ × 6H_2_O) and a sodium acetate buffer solution (pH = 3.6) were also acquired from Sigma-Aldrich. For the microbiological assay, the tryptone soy agar (TSA) ready-to-use preparation was purchased from Oxoid (UK). To conduct the cytotoxicity assay, the Opti-MEM medium with GlutaMAX was purchased from Thermo Fisher Scientific Inc. (USA), while fetal bovine serum was obtained from Gibco (UK). Penicillin, streptomycin, trypsin/EDTA, 3-(4,5-dimethyl-2-thiazolyl)-2,5-diphenyl-2H-tetrazolium bromide (MTT) and dimethyl sulfoxide (DMSO) solutions were from Sigma-Aldrich. Phosphate-buffered saline (PBS) was prepared at the Hirszfeld Institute of Immunology and Experimental Therapy, Polish Academy of Sciences. The eBioscience Annexin V Apoptosis Detection Kit APC was purchased from Invitrogen (Thermo Fisher Scientific Inc.). All reagents were of analytical grade or better. Re-distilled water was used throughput.

### High-throughput reaction-discharge system dedicated to continuously treating apple juice

Direct current atmospheric pressure glow discharge (dc-APGD) generated in contact with a flowing liquid cathode (FLC) was used as the NTAP source, intended for the treatment of AJ. FLC-dc-APGD was ignited in a continuous-flow reaction-discharge system^13,15^. In this reaction-discharge system, the discharge was stably operated in a 3.0–5.0 mm discharge gap, situated between the surface of analyzed AJ, being the FLC, and a sharpened tungsten electrode, being the anode. AJ was delivered to the reaction-discharge system via a quartz capillary (OD = 4.0 mm), onto which a graphite tube (OD = 6.0 mm) was placed. A four-channel peristaltic pump (Masterflex L/S, Cole-Parmer, USA) was used, while AJ was pumped at a flow rate of 2.0–6.0 mL min^−1^. An high-voltage of 1100–1300 V was supplied to the NTAP-based reaction-discharge system using a dc-high-voltage-supply (Dora Electronics Equipment, Poland). Its positive voltage and ground cables were connected to the anode and FLC and the anode, respectively. The discharge current in the enclosed circuit was changed within a 30–50 mA range, and was stabilized by a ballast resistor. Immediately after the exposure of an analyzed AJ sample to FLC-dc-APGD, plasma-treated AJ was collected to polypropylene vials (Sarstedt, Germany) for further studies.

### Multiparameter optimization by the design of experiments and the response surface methodology

To find appropriate experimental conditions to treat AJ by NTAP in the proposed continuous-flow reaction-discharge system, optimization experiments based on the Box-Behnken response surface design were carried out according to the run order given in Table 1. This response surface design included 15 randomized treatments at three different levels of studied experimental parameters, i.e., the flow rate of the FLC (F, in mL min^−1^), the discharge gap (d, in mm) and the discharge current (I, in mA). All uncoded (and coded in brackets) settings of mentioned operating parameters within treatments are given in Table 1 as well. The response of the design was the maximum A (in a. u.) of the most prominent absorption band in the UV/Vis spectrum of AJ at the λ_max_ of 284 nm, which is highly indicative to hydroxybenzoic acids, hydroxycynnamic acids, flavonols, anthocyanidis, and other phenolic substances like tannins^16,17^, as well as the T (in °C) of this juice. Ranges of experimental parameters were selected based on a stable operation of dc-APGD operated in contact with AJ (being the FLC). To evaluate precision of the applied Box-Behnken response surface design, three center points were included. Considering values of measured responses at these points, this precision was high, i.e., 4.0% for the mean A value of 1.01 and 1.0% for the mean T value of 40.1 °C. All treatments within the Box-Behnken design were carried out in one block. Experiments were carried out as follows: portions of AJ were introduced into the flow-through NTAP-based reaction-discharge system, sustained and operated under subsequent parameters settings given in Table 1. NTAP-treated AJ at given experimental conditions was collected and its t was immediately measured after which its UV/Vis absorption spectrum was acquired within next 30 min for a 20-fold diluted sample in the spectral range from 190 to 1100 nm to get the A of the most prominent absorption band at the λ_max_ of 284 nm. All data collected for both responses were fitted with full quadratic functions, comprising linear (F, d, I) and square (F^2^, d^2^, I^2^) effects of examined parameters as well as their two-way interactions (Fd, FI, dI). General equations of functions fitting both responses looked as follows: A = a_0_ + a_1_F + a_2_d + a_3_I + a_11_F^2^ + a_22_d^2^ + a_33_I^2^ + a_12_Fd + a_13_FI + a_23_dI and T = b_0_ + b_1_F + b_2_d + b_3_I + b_11_F^2^ + b_22_d^2^ + b_33_I^2^ + b_12_Fd + b_13_FI + b_23_dI, where a_0_—a_33_ and b_0_—b_33_ are appropriate regression coefficients. To find all statistically significant terms in these regression models for A and t, the backward elimination of terms algorithm was used at the significance level of 90% (α = 0.1), maintaining the hierarchy of terms. Reliability of response surface regression models was established by using the analysis of variance (ANOVA) test of residuals between the measured data and the data modeled by these regression models under all treatments included in the Box-Behnken design. Accordingly, goodness-of-fit of regression models was shown by coefficients of determination (R^2^) and adjusted R^2^ values. In addition, p-values assessed for models and all terms included in these models were given to indicate their statistical significance at α = 0.1. Residuals obtained for both regression models were also visually analyzed according to probability plots (used to verify that residuals were normally distributed) and scatter plots of standardized residuals *versus* the run order (to verify that residuals were independent from each other). Finally, using response surface regression models, optimal parameters settings were selected on the basis of strictly defined individual desirability functions, i.e., d_A_ and d_T_, and the value of the composite desirability function, i.e., D = (d_A_ × d_T_)^1/2^. It was specified that the d_A_ would reach the value of 1 when the A of the absorption band at the λ_max_ in the UV/Vis absorption spectrum of NTAP-treated AJ would have the targeted value of 0.897 (corresponding to untreated AJ). The d_T_ = 1 when the T of NTAP-treated AJ would be maximal but not higher than 50 °C. Both optimal responses were selected so as not to degrade the chemical composition of AJ but to prolong its shelf life and biological safety by profiting from antimicrobial effects of the high temperature treatment.

**Table 1:** The Box-Behnken design matrix, showing the standard order, the run order, the level of analyzed parameters, and responses of the system.

Standard order	Run order	F, mL min^−1^	d, mm	I, mA	A, a. u	T, °C
5	1	4.0 (0)	3.0 (− 1)	30 (− 1)	1.72	41.0
7	2	4.0 (0)	5.0 (+ 1)	30 (− 1)	1.01	35.2
9	3	2.0 (− 1)	3.0 (− 1)	40 (0)	1.53	32.0
12	4	6.0 (+ 1)	5.0 (+ 1)	40 (0)	0.865	39.6
2	5	2.0 (− 1)	4.0 (0)	50 (0)	1.56	30.0
3	6	6.0 (+ 1)	4.0 (0)	30 (− 1)	0.847	35.6
13	7^a^	4.0 (0)	4.0 (0)	40 (0)	0.971	39.6
8	8	4.0 (0)	5.0 (+ 1)	50 (+ 1)	1.12	34.9
6	9	4.0 (0)	3.0 (− 1)	50 (+ 1)	1.19	39.7
11	10	2.0 (− 1)	5.0 (+ 1)	40 (0)	1.37	30.1
10	11	6.0 (+ 1)	3.0 (− 1)	40 (0)	0.950	42.8
4	12	6.0 (+ 1)	4.0 (0)	50 (+ 1)	0.956	43.6
15	13^a^	4.0 (0)	4.0 (0)	40 (0)	1.01	40.4
14	14^a^	4.0 (0)	4.0 (0)	40 (0)	1.05	40.2
1	15	2.0 (− 1)	4.0 (0)	30 (− 1)	1.26	33.0

Studied parameters and responses are labeled as follows: *F* the flow rate of the FLC, *I* the discharge current, *d* the discharge gap, *A* the absorbance, *T* the temperature.

^a^Center points. The treatments carried out at the following settings of the parameters: F (in mL min^−1^) = 4.0 (0), d (in mm) = 4.0 (0) and I (in mA) = 40 (0).

### Identification of reactive oxygen and nitrogen species

ROS and RNS produced during the NTAP treatment of AJ were identified by using optical emission spectrometry (OES). Emission spectra of FLC-dc-APGD were acquired in the range from 200 to 900 nm with a Shamrock SR-500i instrument supported by a Newton DU-920P-OE CCD camera (Andor, UK), during continuously introduction of AJ to the NTAP-based reaction-discharge system by using a peristaltic pump. The spectrometer was operated following the set of optimal operating conditions recommended by a manufacturer.

### Element analysis

An Agilent (USA) ICP OES instrument, model 5110, was used for determining total concentrations of major (Ca, K, Mg, Na), minor (Al, B, Fe, Mn, Sr), and trace (Ba, Cd, Co, Cr, Cu, Ni, Zn) elements in NTAP-treated and untreated AJ. The instrument was operated using the following settings: the RF power, 1.2 kW; the argon plasma flow rate, 12.0 L min^−1^; the argon auxiliary flow rate, 1.0 L min^−1^; the argon nebulizer flow rate, 0.7 L min^−1^; the plasma observation mode, SVDV; the sample delay time, 10 s; the stabilization time, 10 s; the read-out time, 5 s (3 replicates); and analytical lines, Al I 396.2 nm, B I 249.8 nm, Ba II 455.4 nm, Ca II 396.8 nm, Cd II 214.4 nm, Co II 238.9 nm, Cr II 267.7 nm, Cu I 327.4 nm, Fe II 238.2 nm, K I 766.5 nm, Mg II 279.6 nm, Mn II 257.6 nm, Na I 589.6 nm, Ni II 231.6 nm and Zn I 213.8 nm, where I and II denote atomic and the ionic emission lines, respectively. A double-pass cyclonic spray chamber with a SeaSpray concentric nebulizer were used to introduce sample solutions of NTAP-treated and untreated AJ into the instrument. A simple procedure was used to prepare these sample solutions and it included a fivefold dilution of AJ samples along with acidification of resulting sample solutions with HNO_3_ to 1.0 mol L^−1^. Concentrations of Al, B, Ba, Cd, Co, Cr, Cu, Fe, Mn, Ni and Sr were directly measured in such prepared sample solutions. In case of concentrations of Ca, K, Mg and Na, they were additionally twofold (Na), 20-fold (Ca, Mg) and 200-fold (K) diluted. For the calibration, simple standard solutions were used that spanned the concentration range from 0.02 to 2.00 mg L^−1^. Final results were means for three independent samples (n = 3) along with respective standard deviations (SDs). To verify trueness of results, samples of untreated AJ were spiked with 1 mg L^−1^ of Al, B and Fe, 0.5 mg L^−1^ of Mn and Sr and 0.1 mol L^−1^ of Ba, Cd, Co, Cr, Cu and Ni. In case of Ca, K, Mg and Na, the original sample solutions were appropriately diluted and then, resulting ones were spiked with 1.0 mg L^−1^ of these elements.

### Physicochemical analyses

Physiochemical properties of NTAP-treated and untreated AJ were evaluated to interpret the impact of the NTAP treatment on studied AJ, including several features *i.e.*, preservation of the structure of organic compounds, the content of saccharides, color, the content of total phenolics, and the FRAP.

To estimate the effect of the NTAP treatment on the structure of included organic compounds, attenuated total reflectance Fourier transform-infrared spectroscopy (ATR FT-IR) was applied. A Jasco FT-IR 4700 spectrometer (Japan) was used for this purpose and ATR FT-TR spectra were acquired in the spectral range from 4000 to 400 cm^−1^, with resolution of 4 cm^−1^ and 64 scans for each measurement. One drop of analysed samples of NTAP-treated and untreated AJ was placed onto a diamond prism in an ATR accessory. To establish the effect of the NTAP treatment on the content of saccharides, the °Brix scale was employed, measuring the refractive index (RI) of analyzed samples of NTAP-treated and untreated AJ. Initially, samples were 20-fold diluted and then their IR was measured by using a Mettler-Toledo REFRACTO 30PX digital refractometer (USA). Results for NTAP-treated and untreated AJ were given in the ^o^Brix scale, considering the dilution factor and using ICUMSA charts^18^.To check the influence of the FLC-dc-APGD treatment on color of AJ, the colorimetric assay according to the CIE L^*^a^*^b^*^-based system was performed. The CIE L^*^a^*^b^*^ system defines color as differences between light/dark (L^*^), red/green (a^*^) and blue/yellow (b^*^) that set together in a cube-root function gives a color change as an $\Delta$E value^19,20^. A 4-Wave WR-18 colorimeter (Eplaneta, Poland), with a 40 mm measuring head, a D65 illuminator and a 2° standard observer, was used for that purpose. Colorimetric measurements were carried out as follows: 20-fold diluted NTAP-treated or untreated AJ samples were introduced into a measuring cell. Then, measurements were performed, taking L^*^a^*^b^*^ parameters of distilled water as reference values. To study changes in the total content of phenolic compounds, the Folin-Ciocalteu test was applied^21^. In this test, 2.5 mL of the tenfold diluted Folin-Ciocalteu reagent was mixed with 0.5 mL of 20-fold diluted NTAP-treated or untreated AJ samples, and with 2.0 mL of a 7.5% (m/v) Na_2_CO_3_ solution. Then, resulting sample solutions were incubated at 50 °C for 15 min in a water bath (WSL, Poland) and then, cooled down in an ice-bath for 5 min. The A of such sample solutions was measured at 765 nm by using a Specord 210 Plus UV/Vis absorption spectrophotometer (Analytik Jena, Germany). The instrument was zeroed using a respective blank solution. The total content of phenolic compounds in samples of AJ (the mean value for n = 3) was determined based on the calibration curve of gallic acid (0–100 mg L^−1^) and expressed in mg of the gallic acid equivalent (GAE) per L. To establish the effect of the NTAP treatment on the FRAP value, a modified Benzie and Strain protocol was used^22^. In this assay, 100 μL of 100-fold diluted NTAP-treated or untreated AJ samples were mixed with the FRAP reagent (a mixture of TPTZ in a 0.1 mol L^−1^ HCl solution with a 20 mmol L^−1^ FeCl_3_ solution, buffered with a 0.3 mol L^−1^ sodium acetate solution). Then, resultant sample solutions were incubated at 37 °C in a water bath for 4 min (WSL, Poland). Afterwards, the A value of these solutions was measured at 593 nm using the abovementioned Specord 210 Plus UV/Vis absorption spectrophotometer. As before, the spectrophotometer was zeroed using a respective blank solution. As a standard for the FRAP assay, a 10 mmol L^−1^ FeSO_4_ solution was applied.

### Microbiological analyses

#### Microbiological contaminations

At first, 100 µL of either filtered or unfiltered AJ was plated on the Trypticase Soy Agar (TSA; Oxoid, UK) rich medium to examine whether the number of obtained colony forming units (CFUs) could be affected by the filtration procedure. Furthermore, in order to evaluate whether the NTAP-treatment might lead to any microbiological contamination of the final product, 100 µL of NTAP-treated and untreated AJ were plated on the TSA rich medium. All plates were incubated at 37 °C and colonies were counted after 72 h post inoculation. This experiment was conducted in three independent repetitions (n = 3) and final results were averaged.

#### Determination of the apple juice shelf life

To assess how the controlled NTAP treatment influenced the shelf life of the resultant product, the standard microbiological culture method was used. 100 µL of analyzed AJ (untreated and unfiltered AJ; untreated and filtered AJ; or filtered and NTAP-treated AJ) was plated on the TSA rich medium. Corresponding samples of the same volume were taken 1, 2, 3, 5, 6, or 12 days after initiation of the experiment. All plates were incubated at 37 °C and then colonies were counted after 72 h post plates inoculation. This experiment was conducted in three independent repetitions (n = 3). All of AJ samples were stored under room temperature.

### Cytotoxic analyses

#### Cell culture conditions and experimental groups

The Caco-2 cell line (ATCC HTB-37), derived from human colonic adenocarcinoma, was obtained from ATCC. The normal human intestinal epithelial cell line FHs 74 Int (ATCC CCL-241) was immortalized according to the previously published method to receive stable cell lines^23^. Both cell lines were used to assess the effect of NTAP-treated and untreated AJ on cell viability. Cells were maintained in an Opti-MEM with GlutaMAX (Thermo Fisher Scientific Inc.) medium, supplemented with a 2% fetal bovine serum (FBS, Gibco, UK), a 100 U mL^−1^ penicillin solution and a 100 µg mL^−1^ streptomycin solution (Sigma-Aldrich) and incubated at 37 °C under 5% CO_2_ atmosphere^15^. After reaching the confluence, cells were routinely passaged by using a 0.05% trypsin/0.02% EDTA (w/v) solution. To evaluate the impact of NTAP-treated AJ on Caco-2 and FHs 74 Int cell lines, cells were incubated with diluted samples of AJ that were either previously subjected to the NTAP treatment or not. In the first step, in vitro experiments were carried out with cells that were exposed to × 1000, × 100, × 25, × 10, × 4 and × 2 diluted NTAP-treated and untreated AJ in the culture medium without the serum. As a control, the culture medium without the serum was used in all experiments. Because of high cytotoxicity for both human cell lines exhibited by × 4 and × 2 dilutions, × 1000, × 100, × 25 and × 10 dilutions of NTAP-treated and untreated AJ were chosen for further analyses.

#### MTT assay

MTT assay was performed according to method described in previously published article^15^. In shortly, cells were seeded at a concentration of 5 × 10^3^ FHs 74 Int cells/well and 5 × 10^3^ Caco-2 cells/well in dedicated for that purpose 96-well plates. To achieve the best conditions for cells grown, the Opti-MEM with GlutaMAX medium, supplemented with a 100 U mL^−1^ penicillin solution and a 100 µg mL^−1^ streptomycin solution, were used. In this medium there was no added the fetal bovine serum. In the next day, the cells were incubated with the analyzed AJ solutions, including NTAP-treated and untreated proper AJ. The dilutions of analyzed AJ solutions were as follows: × 1000, × 100, × 25, and × 10. The dilution was carried out in the culture medium without the serum (Opti-MEM), each group were treated in triplicates. A 100 μL of a 0.4 mg mL^−1^ MTT solution was added after 24 and 72 h to each well of well plate and incubated for 3 h in dark at 37 °C. Then, the MTT solution was replaced by 100 μL of DMSO and incubated at 37 °C for 10 min to dissolve purple crystals. The optical density (OD) value of obtained mixtures was measured at 570 nm, i.e. OD at 570 nm. A Victor 2 multi-function microplate reader (Perkin Elmer, USA) was used for these measurements. To calculate the metabolic activity, a mean value of OD readings acquired in triplicates in three independent experiments was considered.

#### Apoptosis analysis

eBioscience Annexin V Apoptosis Detection Kit APC (Invitrogen, USA) was used to estimate the apoptosis by flow cytometry^15^. The number of studied apoptotic, i.e. late apoptotic, early apoptotic, and necrotic cells by propidium iodide (PI), was assessed. The number of proper apoptotic was compared to control. As control, the cells treated only with the culture medium were applied. To reach this aim, cells were seeded at a concentration of 1 × 10^4^ cells and cultured in the medium containing the fetal bovine serum in the 48-well plates. Then, after achieving the 70–80% confluence, diluted AJ was added to each well. The dilutions of AJ solutions were as follow: × 1000, × 100, × 25 and × 10 diluted. Subsequently, cells were incubated for further 24 and 72 h. After this time, cell culture supernatants and cells were harvested by trypsinization and washed in cold PBS. In the next step, cell pellets were re-suspended in an Annexin-binding buffer. Annexin V was added and incubation, followed by another washing using cold PBS, was performed. Finally, PI was added to each analyzed sample according to the producer’s protocol. By applying FL4 (λ_em_ = 660 nm) and FL2 (λ_em_ = 535 nm) modes, early apoptotic cells (Annexin V positive, PI negative), late apoptotic cells (Annexin V positive, PI positive), necrotic cells (Annexin V negative, PI positive), and alive cells (Annexin V negative, PI negative) were detected^15^. A FACSCalibur flow cytometer (Becton Dickinson, USA) was used to collect data. To estimate the percentage of apoptotic cells, a Flowing Software 2 program was applied. All calculated values were shown as means along with respective SDs (n = 3). Tests were performed in technical duplicates. Results were analyzed through the unpaired t-test, using GraphPad Prism 5 software. The p-values for comparison of all examined groups with their respective control medium groups or untreated AJ to NTAP-treated AJ groups were calculated applying the one-way ANOVA test with the Dunnett's post hoc test.

## Results and discussion

### Multiparameter models development

**Figure 1:**
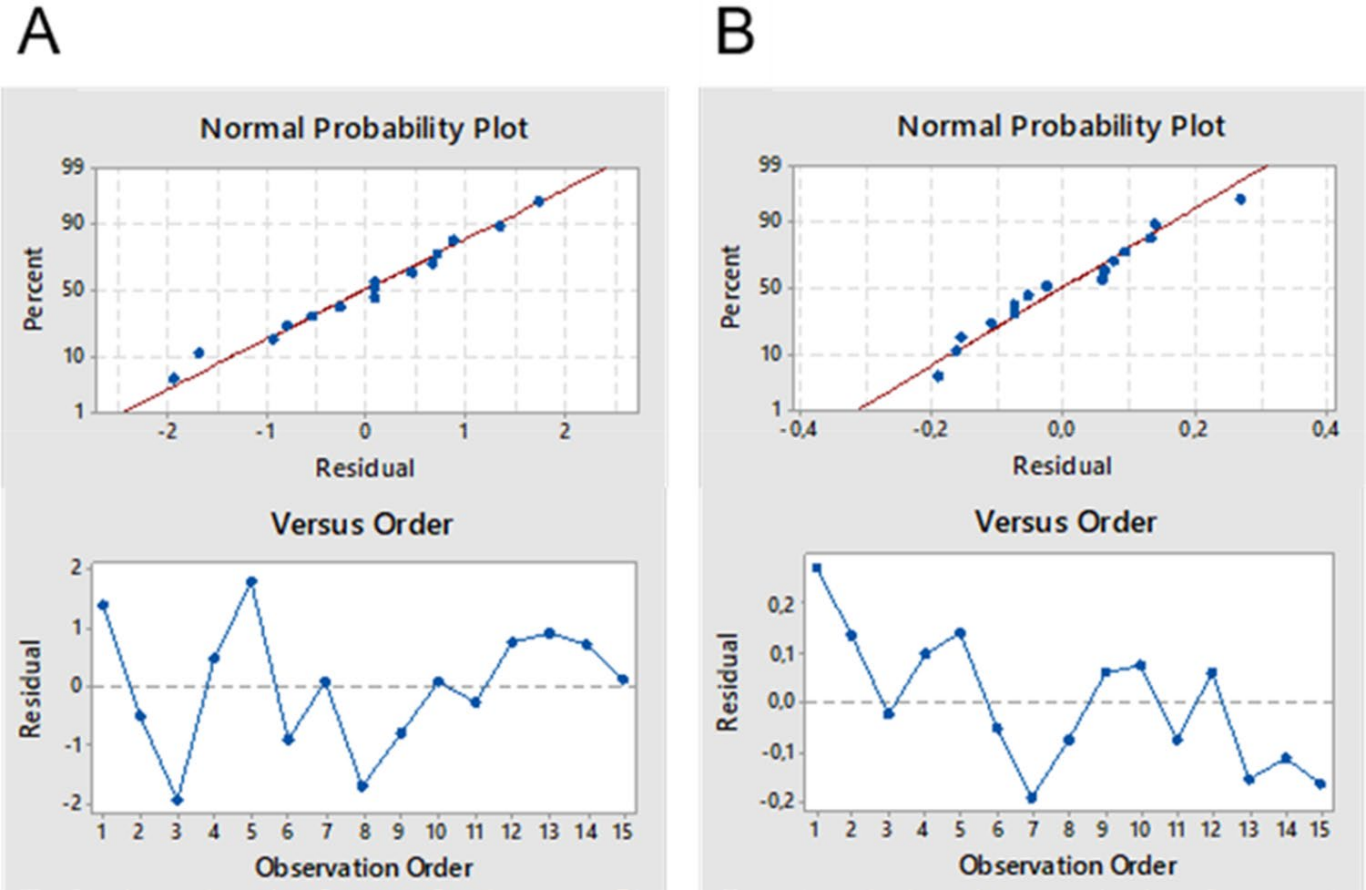
Normal probability plots and scatter plots of standardized residuals versus the observation (run) order for established models for (**A**) the absorbance (A) at the λ = 284 nm in UV/Vis absorption spectra of NTAP-treated apple juice and (**B**) the temperature (t) of this juice.

Application of the FLC-dc-APGD system to treat AJ was aimed at increasing/prolonging its shelf life, but not deteriorating its chemical composition in reference to bioactive compounds affecting its antioxidant properties, *i.e.,* phenolic compounds like hydroxybenzoic acids, hydroxycynnamic acids, flavonols, anthocyanidis and polyphenols^24,25^. The overexposure of apple slices or fresh AJ^26,27^ to the NTAP treatment was previously reported to be responsible for decreasing the content of majority of bioactive compounds. Therefore, appropriate optimization of NTAP treatment conditions is quite important in reference to future applicability of NTAP technology for sterilization and preservation of fruit beverages and other similar products. Herein, the synergic effect of reactive species, *i.e.*, short- and long-lived ROS and RNS, H radicals, electrons and UV radiation^28^, as generated by FLC-dc-APGD, was employed for amelioration of properties of a fresh-like product without degradation of its valuable bioactive compounds after careful optimization of the NTAP system based on the DOE approach according to the Box-Behnken design The response of this system was the A value of the absorption band of NTAP-treated AJ acquired by UV/Vis absorption spectrophotometry and the T of this juice. It was assumed that to preserve nutritional quality of NTAP-treated AJ, the A of the absorption band at 284 nm (Fig. S1) should be at the same level, while to increase its shelf life, the T should be maximized but not higher than 50 °C (see reference no.^2^). Both responses (A, T), gathered according to the Box-Behnken response surface design, were fitted with full quadratic functions. ANOVA tables, summarizing statistical evaluation of established regression functions, are given in Table 2. As can be seen, both models were statistically significant; respective *p*-values for these models were equal to 0.004 and 0.000, respectively, for the A and the T. Corresponding R^2^ values were 76% and 94% and indicated quite good and very good fits of models to the measured data. In addition, values of SD of residuals (S) were relatively low; they were 3.3% and 5.7% of intercepts included in models for the A and the T. Visual analysis of residual plots for both established models proved that they well fitted the experimental data (Fig. 1). Accordingly, all data points followed straight lines in normal probability plots, hence, it was confirmed that residuals were normally distributed. In scatter plots of standardized residuals versus the run order, data points were rather randomly placed on both sides of center lines, indicating that residuals were independent from each other. Hence, the ANOVA statistics of established response surface models and their residuals showed that all the experimental data (A, T) were well approximated with regression equations given in Table 2, and these models could be used for optimization of the NTAP treatment of AJ.

**Table 2:** The output of the analysis of variance (ANOVA) test.

	DF	Adjusted SS	Adjusted MS	F-value^a^	p-value^b^
A = 4.79—1.32⋅10^-1^F—7.72⋅10^-1^d—6.46⋅10^-2^I + 1.62⋅10^-2^dI					
S = 0.1571, R^2^ = 76.30%, R^2^-adjusted = 66.82%					
Model	4	0.7946	0.1986	8.05	0.004
Linear	3	0.6910	0.2303	9.33	0.003
F	1	0.5593	0.5593	22.66	0.001
D	1	0.1317	0.1317	5.34	0.044
I	1	0.0000	0.0000	0.00	0.977
Two-way interactions					
Error	10	0.2468	0.0247		
Total	14	1.0414			
T = 2.42⋅10^1^ + 2.74⋅10^-1^F—1.96d—6.14⋅10^-1^I—7.44⋅10^-1^F^2^—1.40⋅10^-2^I^2^ + 1.38FI					
S = 1.383, R^2^ = 94.58%, R^2^-adjusted = 90.52%					
Model	6	267.234	44.539	23.27	0.000
Linear	3	198.787	66.262	34.62	0.000
F	1	166.531	166.531	87.02	0.000
D	1	30.811	30.811	16.10	0.004
I	1	1.445	1.445	0.76	0.410
Square	2	38.197	19.098	9.98	0.007
F^2^	1	32.916	32.916	17.20	0.003
I^2^	1	7.300	7.300	3.81	0.087
Two-way interactions	1	30.250	30.250	15.81	0.004
F × I	1	30.250	30.250	15.81	0.004
Error	8	15.310	1.914		
Total	14	282.544			

*DF* Degrees of freedom, *SS* Sum of squares, *MS* Mean squares, *S* Standard deviation of residuals representing how far data values fall from fitted values. R^2^ Coefficient of determination showing the percentage of variation in the response that is explained by the model.

^a^Probability measuring the evidence against the null hypothesis.

^b^The test statistics determining association of a given model/term with the response.

### The effect of parameters on responses of the system

**Figure 2:**
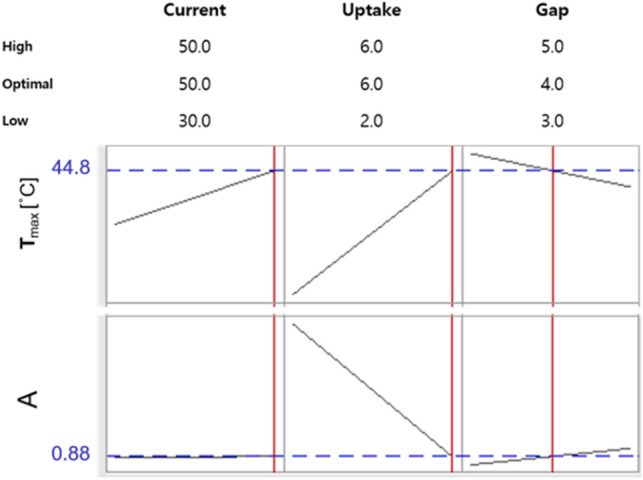
Optimization plots with optimal values for the NTAP treatment of apple juice.

Considering all terms in equations of above-mentioned models and optimization plots (Fig. 2), A and T values were dependent mostly on F and I settings. The increase in the F at given I and d settings resulted in decreasing the A value. It was likely because the contact time of AJ with the gaseous phase of the discharge was getting shorter in these conditions, and hence, eventual destruction of biogenic molecules in AJ seemed to be avoided. Nevertheless, the T value of AJ increased in these conditions possibly as a result of intensification of excitation processes of such molecules as NO, OH, and N_2_. Indeed, it was noted that intensities of heads of the most prominent molecular bands of NO, OH and N_2_ molecules gradually increased when the F was elevated within the studied range. This contributed to rise in rotational temperatures of OH and N_2_ molecules, i.e., T_rot_(OH) and T_rot_(N_2_), as was determined on the basis of comparisons with simulated molecular emission spectra^29^.

To confirm this observation, detailed identification of ROS and RNS produced during the NTAP treatment of AJ was performed by using OES. OES spectra of FLC-dc-APGD are presented in Fig. S2. Generally, numerous NO (the A^2^Σ^+^-X^2^Π γ-system) and N_2_ (the C^3^Π_u_-B^3^Π_g_ second positive system) bands were identified in the spectral range of 200–260 nm and 280–550 nm, respectively. Additionally, two strong OH bands belonging to the A^2^Σ-X^2^Π system, with band heads at 282.9 nm (1–0) and 308.9 nm (0–0) were noted. The NH (0–0) band of the A^3^Π-X^3^Σ^-^ system (with the head at 336.0 nm) was also easily excited. Moreover, atomic lines of H radicals (at 486.1 nm and 656.3 nm) and O radicals (at 777.2, 777.4 and 844.6 nm) were identified. It should be noted, that atomic lines of Na (at 588.9 and 590.0 nm) and K (at 766.5 and 769.9 nm) were also observed. The presence of these metals (Na, K) was likely resulting from thermal evaporation and/or sputtering of AJ. Similar observations were reported by Jamroz and Zyrnicki^29^ or Bruggeman et al*.*^30^, who examined similar atmospheric pressure discharge systems. Concluding, the temperature of the gaseous phase of the NTAP-based system with FLC-dc-APGD increased in these conditions and tended to increase the T value of NTAP-treated AJ introduced at higher F values. The increase in the I at given F and d settings also resulted in rising the T of NTAP-treated AJ. As in case of the F growth, it could be associated with intensification of excitation processes in the gaseous phase of the discharge. Indeed, intensities of band heads of above-mentioned molecular species (NO, OH, N_2_) and intensities of H and O atomic lines in emission spectra of FLC-dc-APGD operated in contact with continuously introduced AJ were established to gradually increase. This contributed to elevation of T_rot_(OH) and T_rot_(N_2_) and the temperature of the gas phase of the analyzed FLC-dc-APGD discharge. Surprisingly, the A value was practically unchanged in these conditions. It could be presumed that in this case, although the concentration of short-lived reactive species like NO^•^, ^•^OH, O_2_^•-^ and O could increase when I settings were elevated, their penetration depth was rather limited in the applied continuous-flow reaction-discharge system, in particular when F settings were also high.

### Optimal conditions for the NTAP treatment of apple juice and models validation

Established response surface regression models enabled to select optimal settings of the F, the d and the I so as to provide the A value of the NTAP-treated apple value being the same as for untreated AJ along with the highest accepted T value. These conditions were presumed to be responsible for maintaining the nutritional value of AJ and prolonging its shelf life. Considering these requirements to be obtained (d_A_ = 1 when the A reached the value of 0.897, and d_T_ = 1 when the T reached a maximum value but not higher than 50 °C), it was established that optimal settings of studied parameters were as follows: F = 6.0 mL min^-1^, d = 4.0 mm, and I = 50 mA. In these conditions, modeled A and T values were 0.897 (d_A_ = 1.00) and 44.8 °C (d_T_ = 1.00), respectively. With these settings, the D value was 1.00. For this combination of parameters settings, both models were validated in the independent experiment. Accordingly, the FLC-dc-APGD system was run under established optimal conditions for the AJ treatment. Then, the T of such NTAP-treated AJ was measured and it was analyzed within 30 min by UV/Vis absorption spectrophotometry. The following results were obtained: the A was 0.897 ± 0.010 (λ = 284 nm) and the T was 47.8 ± 0.6 °C.

### Nutritional quality of NTAP-treated apple juice

To assess the effect of the NTAP treatment of AJ by using the FLC-dc-APGD system, being operated under parameters settings selected by the Box-Behnken response surface design, on its nutritional value, the following features were determined in untreated and treated AJ and compared, i.e., the content of selected elements (Al, B, Ba, Ca, Cd, Cr, Cu, Fe, K, Mg, Mn, Na, Ni, Sr and Zn), changes in the structure of organic compounds present in AJ, the content of saccharides (**°**Brix scale), color stability, the total phenolic content, and the FRAP. Additionally, microbiological properties of NTAP-treated AJ, potentially having the impact on the shelf life of this product, were evaluated. Finally, cytotoxic properties of NTAP-treated AJ were assessed towards the normal human intestinal epithelial cell line-FHs 74 Int and the heterogeneous human epithelial colorectal adenocarcinoma Caco-2 cell line. The applied NTAP treatment of AJ was performed under optimal settings of studied parameters, *i.e.*, F = 6.0 mL min^−1^, d = 4.0 mm and I = 50 mA.

#### Concentrations of selected elements

Concentrations of examined elements in untreated and NTAP-treated AJ are given in Table 3. As can be seen, the NTAP treatment of AJ resulted in elevating their concentrations by 8–10% (Ca, K, Fe, Mg, Na and Sr) and 11–15% (Al, B, Ba, Cu, Mn and Zn). When it was revealed that the NTAP treatment was responsible for water evaporation, which yield was assessed to be 11.0 ± 1.5%, concentrations of elements turned out to be at the same level. Apparently, differences between concentrations of all examined elements in NTAP-treated AJ and their respective concentrations in untreated AJ were statistically insignificant at the 95.0% significance level (α = 0.05). Calculated values of the two-sample t-test used for this comparison were lower than the appropriate critical value of this test. As such, the NTAP treatment of AJ in the described continuous-flow reaction-discharge system indeed did not lead to deterioration of its nutritional value in reference to the content of selected major, minor and trace elements. It is also worth to notice that water will be evaporated in NTAP-treated AJ so the controlled NTAP-treatment of AJ could lead to increase the concentration of all elements.

**Table 3:** The total concentration of Al, B, Ba, Ca, Cd, Cr, Cu, Fe, K, Mg, Mn, Na, Ni, Sr, Sr, and Zn in the untreated and NTAP-treated apple juice.

Element	Concentration, mg L^−1^		Increase, %	F_calc._^b^	t_calc._^c^	Recovery^d^, %
	Untreated	NTAP-treated/NTAP-treated^a^				
Al	0.662 (3.6%)	0.746/0.660 (2.0%)	12.7	3.22	+ 0.123	96.6 (2.2%)
B	2.60 (1.3%)	2.91/2.59 (1.5%)	11.9	1.32	− 0.340	102.1 (3.2%)
Ba	0.096 (2.2%)	0.108/0.100 (1.9%)	12.5	1.34	+ 0.074	97.5 (1.9%)
Ca	71.2 (1.7%)	77.3/68.80 (3.2%)	8.6	3.31	− 1.657	98.0 (2.7%)
Cd	< 0.005	< 0.005	–	–	–	94.8 (2.4%)
Cr	< 0.009	< 0.009	–	–	–	92.7 (3.6%)
Cu	0.054 (4.3%)	0.061/0.050 (3.8%)	13.0	1.27	+ 0.162	95.9 (4.2%)
Fe	0.869 (6.6%)	0.959/0.854 (6.4%)	10.4	1.10	− 0.339	101.5 (2.5%)
K	860 (4.5%)	927/825 (7.7%)	7.8	2.69	− 0.814	99.1 (1.2%)
Mg	47.6 (1.0%)	52.3/46.6 (2.1%)	9.9	4.22	− 1.678	98.3 (2.2%)
Mn	0.342 (1.4%)	0.384/0.342 (1.6%)	12.3	1.30	− 0.057	96.3 (2.9%)
Na	17.7 (1.5%)	19.2/17.1 (3.0%)	8.5	3.73	− 1.836	105.3 (4.7%)
Ni	< 0.003	< 0.003	–	–	–	94.1 (2.0%)
Sr	0.202 (1.3%)	0.222/0.198 (1.9%)	9.9	2.04	− 1.671	99.3 (1.2%)
Zn	0.082 (2.6%)	0.094/0.084 (1.8%)	14.6	2.00	+ 1.102	97.6 (2.8%)

The average concentrations (n = 3) along with the relative standard deviations (RSDs) in brackets.

^a^The water evaporation rate was included (11.0 ± 1.5%).

^b^The two-sample F-test was used at α = 0.05. F_crit._ (α = 0.05, df_1_ = 2, df_2_ = 2) = 8.78. Non-statistically significant differences are underlined.

^c^The two-sample t-test was used at α = 0.05. t_crit._ (α = 0.05, df = 3 + 3–2 = 4) = 2.78. Non-statistically significant differences are underlined.

^d^The average values (n = 3) along with the standard deviations (SDs) in brackets.

#### Physicochemical properties of NTAP-treated apple juice

To establish the effect of the controlled FLC-dc-APGD treatment on physicochemical properties of analyzed AJ, analyses related the organic compounds structure, the saccharides content (˚Brix scale), color, the total polyphenolics content, and the FRAP were performed for NTAP-treated and untreated AJ.

To determine the content of organic compounds, ATR FT-IR spectroscopy was used. As can be seen from Fig. S3, O–H, N–H, C=O, and C–O stretching vibrations were identified and associated with bands at 3276, 2358, 1634 and 1057 cm^−1^ in spectra of untreated AJ, respectively. The occurrence of these bands was related to the presence of carbohydrates and polyols^31,32^. As was suspected, all organic compounds identified in untreated AJ were also present in NTAP-treated AJ since intensities and shapes of vibration bands were comparable. Similar observations were noticed for the content of saccharides, based on the calculated ˚Brix value. In this case, the ˚Brix value was 13.1° and 13.0° for untreated and NTAP-treated AJ, respectively. This clearly confirmed that the content of saccharides in AJ did not change during its NTAP treatment. These results are slightly different from those reported by Liao et al.^4^ and Vukic et al.^33^, who found that the treatment of proper juice by DBD was responsible for its increased ˚Brix value. However, it should be pointed out that the applied NTAP was dc-APGD generated in contact with analyzed AJ that acted as the FLC of the discharge system. As compared to a point treatment of very small amounts (3 mL) of AJ on a Petri dish with the aid of a DBD jet, reported by Liao et al.^4^ and to 100 mL of either orange or carrot juice on a liquid container^33^, the proposed flow-through reaction-discharge system enabled to constantly treat the whole flow of AJ because its surface, being in contact with the gas phase of the discharge, was instantly replenished by fresh portions of this juice. In such a case, an unlimited amount of AJ could be treated in a relatively short time, only related to the flow rate of AJ.

To reveal the influence of the applied NTAP system on color of analyzed AJ, colorimetric measurements were carried out for both untreated and NTAP-treated AJ. These measurements were carried out by using distilled water as the reference sample. The difference in color (defined by ∆E) between water and untreated and NTAP-treated AJ was 1.448 ± 0.02 and 1.456 ± 0.01, respectively. The difference was just 0.008 and fitted into SD of measurements. Hence, it was concluded that the NTAP treatment did not change color of analyzed AJ. Up to now, there were several studies associating the impact of different NTAPs on color of AJ^2–4,7^. In these works, the established color measure was either decreased^2–4^ or increased^7^, which might be related to the type of the applied NTAP source and the treatment time. Here, color of AJ did not change, however, it should be emphasized that the continuous-flow reaction-discharge system, *i.e.*, FLC-dc-APGD, was applied as the NTAP source.

The impact of NTAP on the total content of phenolics in AJ was assessed to reveal its antioxidant capacity^3^. It was found that the controlled NTAP-treatment of AJ caused the increase of the total content of phenolic compounds by 11% in contrast to untreated AJ (Table 4), taking into account water evaporation from AJ during the process. These results are in agreement with results reported by Dasan and Boyaci^2^, Illera et al*.*^3^, and Liao et al*.*^4^. As was suggested by these authors^2–4^, elevation in the total content of phenolics after the NTAP treatment of AJ could be related with a breakdown of cellular membranes of the dissolved plant material, resulting in releasing additional polyphenolic compounds.

**Table 4:** The total content of phenolics and the FRAP value of untreated and NTAP-treated apple juice (n = 3).

	Untreated	NTAP-treated/NTAP-treated^a^	Increase, %	F_calc._^b^	t_calc._^c^
Total content of phenolics, mg per L of GAE	167.6 (3.5%)	205.6/185.4 (1.2%)	22.7	6.36	+ 4.941
FRAP, mmol of Fe(II) per L	7.07(5.9%)	7.93/7.14 (3.8%)	12.2	2.42	+ 0.243

The average concentrations (n = 3) along with the relative standard deviations (RSDs) in brackets.

^a^The water evaporation rate was included (11.0 ± 1.5%).

^b^The two-sample F-test was used at α = 0.05. F_crit._ (α = 0.05, df_1_ = 2, df_2_ = 2) = 8.78. Non-statistically significant differences are underlined.

^c^The two-sample t-test was used at α = 0.05. t_crit._ (α= 0.05, df = 3 + 3–2 = 4) = 2.78. Non-statistically significant differences are underlined.

Considering the impact of NTAP on the FRAP value of AJ, it was noticed that the applied continuous-flow reaction-discharge system, i.e., FLC-dc-APGD, did not affect the FRAP of analyzed AJ (Table 4). However, it should be emphasised that the water evaporation rate of 11% noted for NTAP-treated AJ was taken into account for this calculation.

#### Microbiological properties of the NTAP-treated apple juice

To analyze potential variation in microbiological properties of analyzed AJ, standard plating assays on rich growth media that involved untreated and NTAP-treated AJ samples were carried out. As no CFUs per mL were noticed in untreated and NTAP-treated AJ, it was confirmed that the controlled treatment of AJ by NTAP did not lead to any microbiological contamination that could result from the additional processing of the product. In other words, NTAP-treatment was carried out under sterile conditions, eliminating the risk of potential contamination e.g., from construction parts of the reaction-discharge system or air. NTAP-treated AJ exhibited identical microbiological properties as freshly opened AJ. Besides, the applied filtration process did not either affect the number of observed CFUs mL^−1^ in filtered AJ. Hence, it was concluded that filtration did not change microbiological properties of studied AJs.

The influence of the controlled NTAP treatment on the shelf life of AJ was assessed by using the standard plating assays as well. Three experimental groups were included i.e., untreated and unfiltered AJ, untreated and filtered AJ, and filtered and NTAP-treated AJ. It was found that up to 6 days post initiation of the study, all samples from analyzed AJ were free from any microbiological contamination (0 CFU mL^−1^; see Table 5). First evidence for contamination of AJ was collected after 12 days in case of untreated AJ (see Table 5). For NTAP-treated AJ, still no microorganisms were spotted at that time. Basing on presented results, it was concluded that the NTAP treatment led to prolongation of the shelf life of AJ. Similar outcomes were reported in previous studies^2,4–8^, but AJ samples were artificially inoculated with bacterial strains in these works before being treated by NTAPs. Hence, it was suggested that antimicrobial properties of the continuous-flow reaction-discharge NTAP system used here could also be associated with efficient production of ROS and RNS, including NO, N_2_, OH, NH, H and O radicals (as identified by OES, see Fig. S2 for more details). According to Kilonzo-Nthenge et al*.*^9^, generated ROS and RNS might efficiently eradicate bacterial vegetative cells. Therefore, NTAP-treated products avoid undesirable microbiological contaminations and exhibit the longer shelf life, while the FLC-dc-APGD continuous-flow reaction-discharge system particularly seemed to be convenient to refine and ameliorate large amounts of AJ and other juices in a controlled way.

**Table 5:** The number of colony forming units per mL in analyzed AJ. Samples were taken 12 days post initiation of the experiment.

Repetition	Group A: untreated and unfiltered AJ	Group B: untreated and filtered AJ	Group C: filtered and NTAP-treated AJ^a^	Negative control^b^
I	20 CFU mL^−1^	30 CFU mL^−1^	0 CFU mL^−1^	0 CFU mL^−1^
II	30 CFU mL^−1^	30 CFU mL^−1^	0 CFU mL^−1^	0 CFU mL^−1^
III	30 CFU mL^−1^	40 CFU mL^−1^	0 CFU mL^−1^	0 CFU mL^−1^

^a^FLC-dc-APGD treatment conditions: F = 6.0 mL min^-1^, d = 4.0 mm, I = 50 mA.

^b^Negative control: TSA plates incubated at 37 °C for 72 h.

#### Cytotoxic properties of the NTAP-treated apple juice

The human intestinal Caco-2 cell line model was extensively used over past few years to screen bioavailability of elements ions from various food products and beverages^10,34^. Here, the cytotoxic effect of NTAP-treated AJ on human colon adenocarcinoma cells (Caco-2) and normal human intestinal epithelial cells (FHs 74 Int) were studied by using the MTT assay in order to reveal any cytotoxic properties of NTAP-treated AJ.

**Figure 3:**
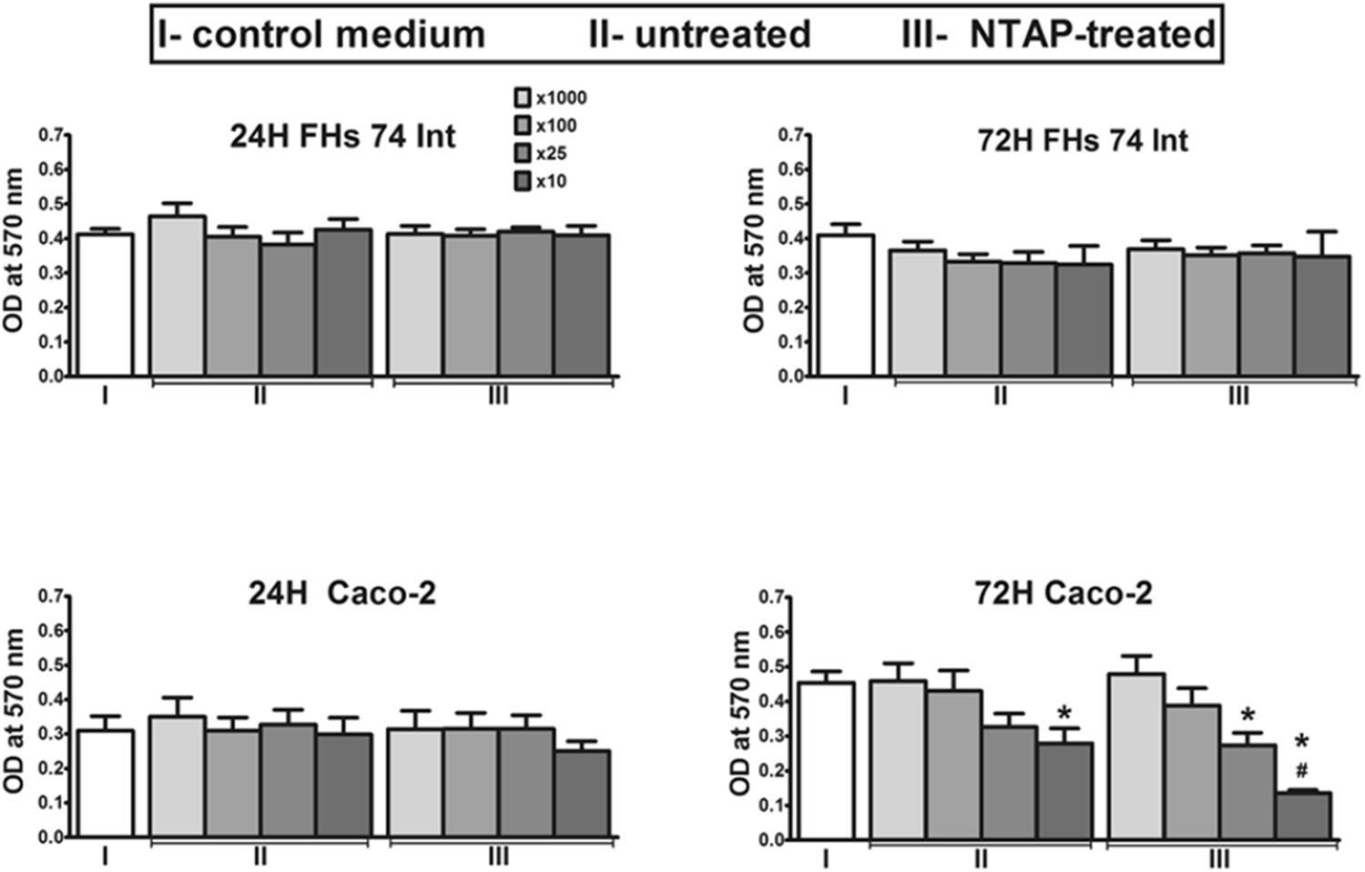
Proliferation measured as the optical density at 570 nm of the human intestinal epithelial cell line (FHs 74 Int) and the human colorectal adenocarcinoma cell line (Caco-2) after exposure to untreated and NTAP-treated apple juice. Dilutions of apple juice samples ranged from × 1000 to × 10. Incubation was performed for 24 and 72 h. The data are shown as mean ± SEM values for three analyses done in triplicate. Statistical comparison was performed by using ANOVA with Dunnett's post hoc tests and compared to the control medium group (cells treated with the medium alone); *p < 0.05 or to untreated apple juices, ^#^p < 0.05.

It was found that NTAP-treated AJ did not inhibit the growth of both human cell lines after 24 h of observation (Fig. 3). When NTAP-treated AJ was used (× 10 dilutions), only limited growth inhibition for Caco-2 cells was observed, however; the result was not statistically significant. Further observations revealed no cytotoxicity effects towards untreated or NTAP-treated AJ in the normal intestinal cell line even after 72 h. Simultaneously, untreated AJ (× 10 dilutions) and NTAP-treated AJ (× 25 and × 10 dilutions) induced strong inhibition of Caco-2 cells proliferation after 72 h as compared to the control (p < 0.05) (Fig. 3). Moreover, the controlled NTAP treatment of AJ (after 72 h) changed cellular responses for tumour cells. Caco-2 cells viability was lower for the NTAP-treated AJ (× 10 dilutions) *versus* untreated AJ (× 10 dilutions) (p < 0.05). These results suggested that the NTAP treatment had no harmful effect on nutritional properties of AJ towards normal cells and preserved its bioactive components from any degradation. Similar results were previously reported for AJ exposed to UV action, however, in these studies UV-treated AJ revealed no cytotoxic effect on normal intestinal cells but exhibited the significantly induced inhibitory effect on the growth of human colon cancer cells^35^. On the other hand, the data published by Kowalczewski et al.^36^ indicated that pure potato juice exhibited selective cytotoxic activity against cancer and normal colon cells.

**Figure 4:**
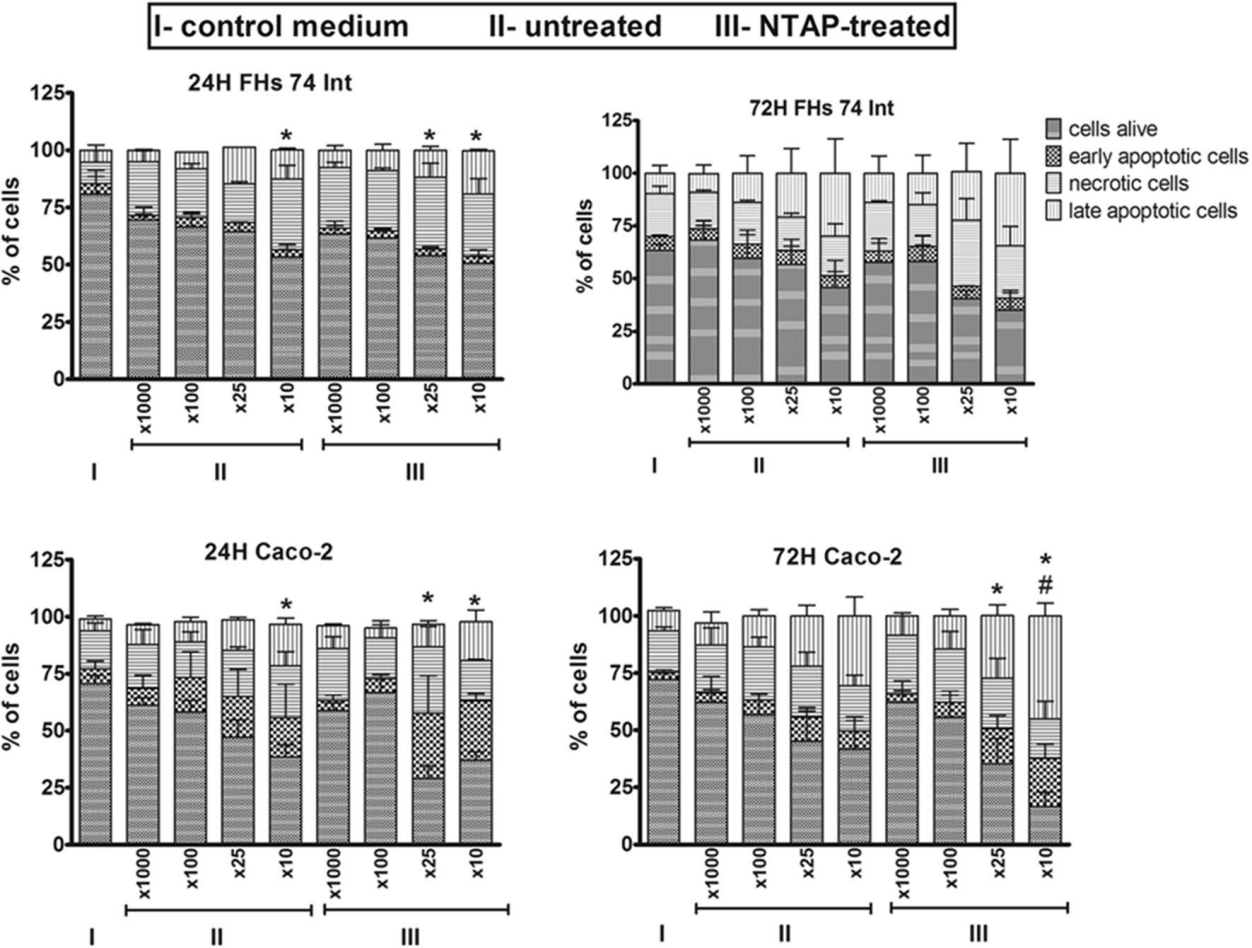
Percentages of apoptotic cells. Human intestinal epithelial cells (FHs 74 Int) and human colorectal adenocarcinoma cells (Caco-2) after exposure to untreated and NTAP-treated apple juice in dilutions ranging from × 1000 to × 10. Incubation was performed for 24 and 72 h. The treatment with the culture medium was applied as the control. Percentages of alive cells, early apoptotic cells, late apoptotic cells and necrotic cells were calculated as mean ± SEM values of three independent experiments, each performed with triplicate wells for each treatment group. Statistical comparison was performed by using ANOVA with Dunnett's post hoc test and juxtaposed to the control medium group (cells treated with the medium alone), *p < 0.05 or to untreated apple juices, ^#^p < 0.05.

In the next step, cytotoxic activity of NTAP-treated AJ (dilutions from × 1000 to × 10) on Caco-2 and FHs 74 Int cells was investigated after 24 h and 72 h (Fig. 4). In general, examination of cytotoxic and apoptotic effects suggested that normal FHs 74 Int cells were more resistant to NTAP-treated AJ in comparison to Caco-2 cancer cells, especially 72 h after application of NTAP-treated AJ. Analyzed AJ decreased viability in both studied cell lines (× 10 dilutions and × 25 dilution) after 24 h (p < 0.05). Moreover, application of untreated AJ (× 10 dilutions) exhibited early cytotoxic effects for both cell lines (p < 0.05) in contrast to the control after 24 h. In addition, for the Caco-2 tumour cells, a significant cytotoxic effect was noted at 72 h after their treatment by NTAP-treated AJ. However, no cytotoxicity to normal FHs 74 Int cells was noted in these conditions. Similar results were obtained by Siu et al*.*^37^, where a transient and significant decline of cell viability was observed at 24 h with NTAP-treated glioma cell line models, followed by reconstitution of cell lines viability by 72 h. Application of NTAP-treated AJ towards studied cell lines mainly increased the number of late apoptotic and necrotic cells (up to 90% for × 10 dilutions of AJ) and to a lesser extent of early apoptotic cells (maximally 30% for × 10 dilutions of AJ) for cancer Caco-2 cells. Taking into account the normal FHs 74 Int cell line, application of NTAP-treated AJ was not cytotoxic after 72 h and observed reduction in the percentage of alive cells was not statistically significant. These results also indicated that NTAP-treated AJ induced cell cytotoxicity for the cancer Caco-2 cell line, as there was a statistically significant difference in the number of alive cells between investigated NTAP-treated AJ groups *versus* untreated AJ, for × 10 dilutions (p < 0.05). Herein presented results clearly show that NTAP-treated AJ might promote antitumor activity. This strategy is probably realized mainly due to augmentation in the concentration of ROS and RNS produced during NTAP operation^38^, as was shown by OES for NTAP-treated AJ (Fig. S2).

## Conclusions

Within the present study it was found that the NTAP treatment of AJ changes its properties. Because there is a variety of experimental factors that might affect physicochemical properties of studied AJ, the DOE approach along with the RSM were applied to carry out multivariate optimization of NTAP-treated AJ production. As a result, optimal operating conditions for the controlled treatment of AJ by the FLC-dc-APGD continuous-flow reaction-discharge system was found and are as follows: the flow rate of AJ = 6.0 mL min^-1^, the distance between electrodes = 4.0 mm and the discharge current = 50 mA. NTAP-treated AJ, obtained under these optimal operating conditions, was analyzed in reference to its nutritional, physicochemical, microbiological, and cytotoxic properties, and compared to those for untreated one. It was revealed that the controlled NTAP treatment of AJ resulted in: (1) increasing the total content of Ca, Fe, K, Mg and Na by 8–10% as well as Al, B, Ba, Cu, Mn and Zn by 11–15%, as well as phenolic compounds by 23%, (2) increasing antioxidant activity of AJ by 12%, (3) extending the shelf life of AJ by 12 days, (4) inducing cell cytotoxicity in the human colorectal adenocarcinoma cells treated by newly ameliorated AJ. It looks that application of NTAP in the form of FLC-dc-APGD for the AJ treatment is a very promising method of its amelioration that could be commonly applied in the future in the industrial sector. Due to the unique design of the discharge-reaction system for FLC-dc-APGD and its inherent nature, i.e. treated AJ is the cathode of the discharge system, normally unlimited amounts of AJ could be treated with NTAP in a relatively short time. This is definitely a clear difference with DBD jets, which operate point wise and could be applied for small sample volumes.

## Supplementary information

Supplementary Information
