# Supplementary material for: Comprehensive studies on the properties of apple juice treated by non-thermal atmospheric plasma in a flow-through system
Source: Sci Rep. 2020 Dec 3;10:21166. doi: 10.1038/s41598-020-78131-6 (PMC7712830; doi:10.1038/s41598-020-78131-6)
Supplement: Supplementary file 1 — Supplementary Information [file 41598_2020_78131_MOESM1_ESM.docx]

**SUPPLEMENTARY MATERIALS**

**Comprehensive studies on the properties of apple juice treated by non-thermal atmospheric plasma in a flow-through system**

Anna Dzimitrowicz^1*^, Aleksandra Bielawska-Pohl^2,^, Pawel Pohl^1^, Piotr Cyganowski^3^, Agata Motyka-Pomagruk^4^, Tymoteusz Klis^1^, Malgorzata Policht^1^, Aleksandra Klimczak^2^, and Piotr Jamroz^1^

^1^ Wroclaw University of Science and Technology, Department of Analytical Chemistry and Chemical Metallurgy, Wybrzeze St. Wyspianskiego 27, 50-370 Wroclaw, Poland

^2^ Hirszfeld Institute of Immunology and Experimental Therapy Polish Academy of Science, Laboratory of Biology of Stem and Neoplastic Cells, R. Weigla 12, 53-114 Wroclaw, Poland

^3^ Wroclaw University of Science and Technology, Department of Polymer and Carbonaceous Materials, Wybrzeze St. Wyspianskiego 27, 50-370 Wroclaw, Poland

^4^ University of Gdansk, Intercollegiate Faculty of Biotechnology University of Gdansk and Medical University of Gdansk, Laboratory of Plant Protection and Biotechnology, Abrahama 58, 80-307 Gdansk, Poland

^*^anna.dzimitrowicz@pwr.edu.pl

**Overview:** In Supplementary Materials we have included additional three Figures associated with the discussed topic. Figure S1 shows UV/Vis absorption spectrum of the apple juice treated by NTAP under optimal operating conditions. Figure S2 presents optical emission spectrum of the direct current atmospheric pressure glow discharge (dc-APGD), being a source of NTAP, which was used for the apple juice treatment. Figure S3 shows the Attenuated Total Reflectance Fourier Transform Infrared Spectroscopy (ATR FT-IR) spectra of the untreated as well as NTAP-treated apple juice.

**
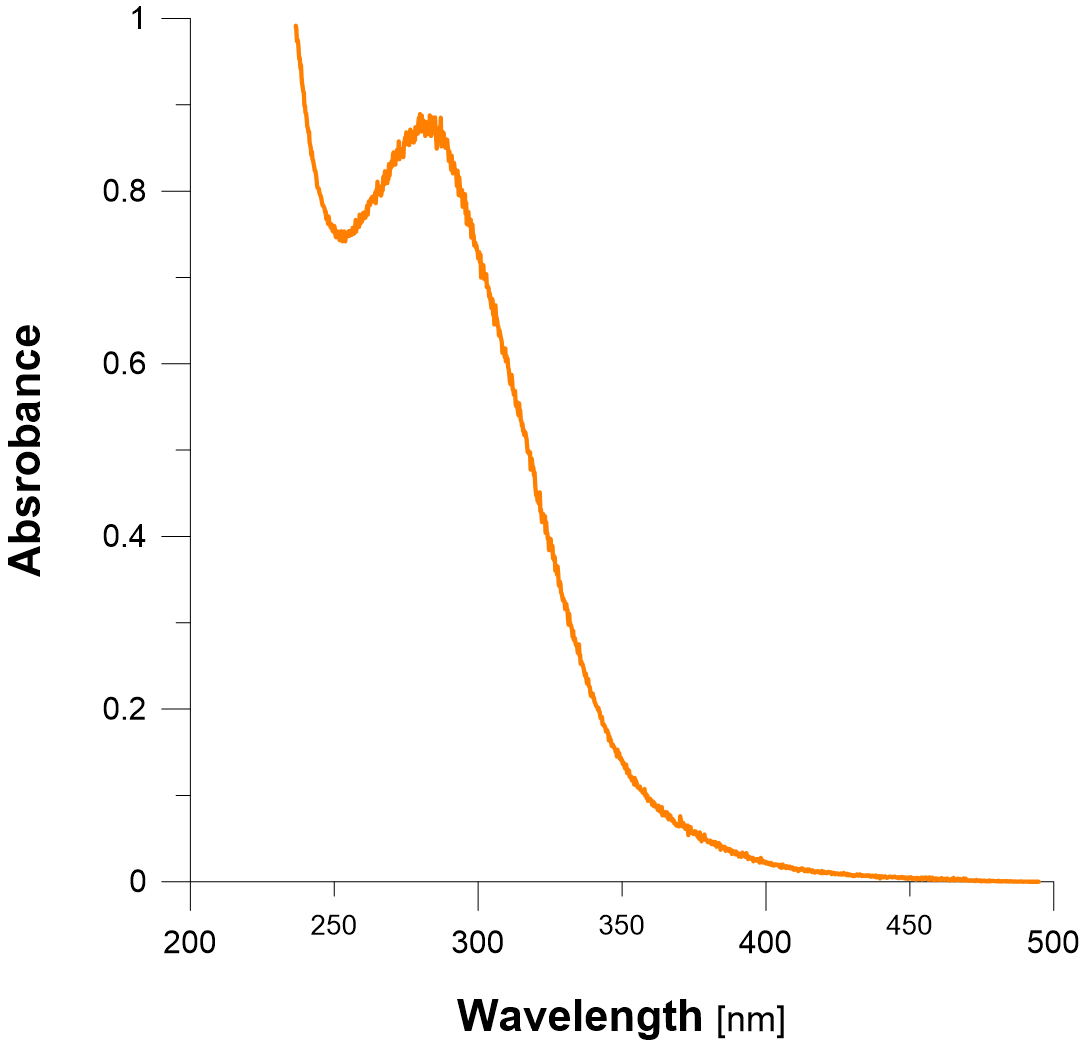
**

**Figure S1.** UV/Vis spectrum of the apple juice sample treated by NTAP under optimal conditions.

***
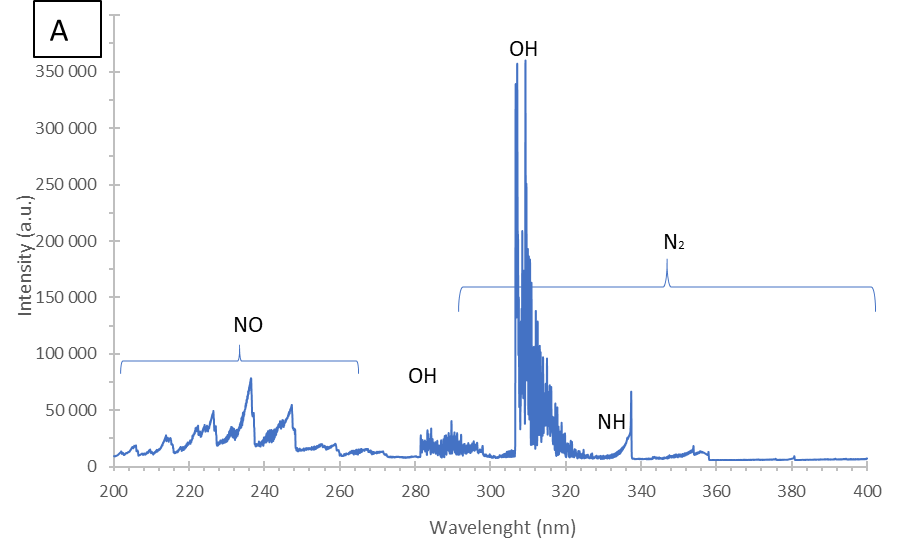

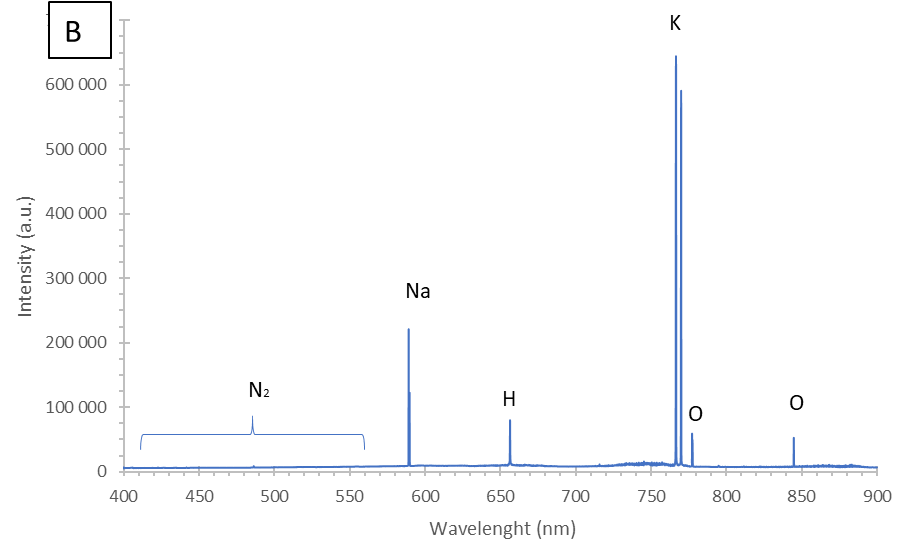
***

**Figure S2.** The optical emission spectra of NTAP, operated in the contact with an apple juice under optimal operating conditions and acquired within a range **(A)** 200-400 nm and **(B)** 400-900 nm.

**
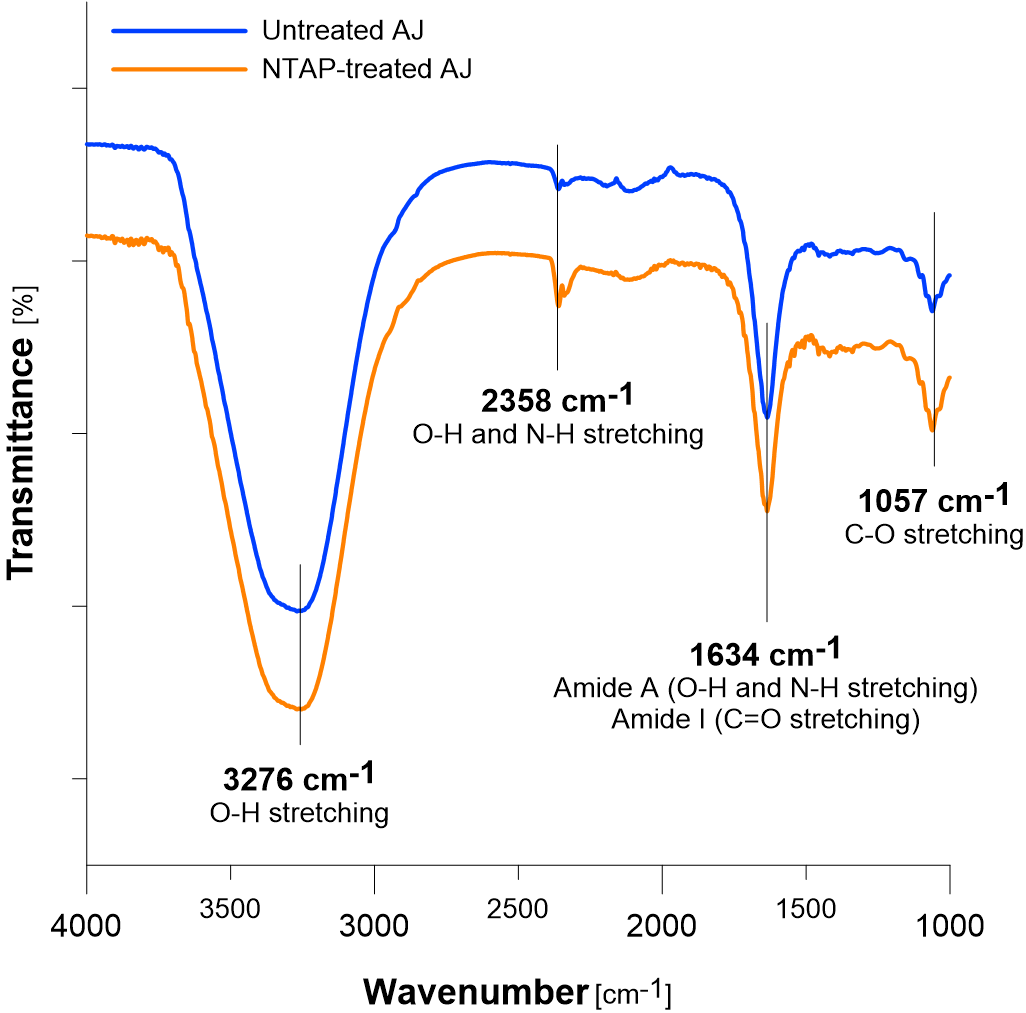
**

**Figure S3.** ATR FT-IR spectra of the untreated as well as NTAP-treated apple juice.
